# GC-MS Analysis of β-Carotene Ethenolysis Products and their Synthesis as Potentially Active Vitamin A Analogues

**DOI:** 10.1080/15376510701623656

**Published:** 2008-06-23

**Authors:** I. Jermacz, J. Maj, J. W. Morzycki, A. Wojtkielewicz

**Affiliations:** Institute of Chemistry, University of Bialystok, Al. Pilsudskiego 11/4, 15-443, Bialystok, Poland

**Keywords:** β-Carotene, Cross-Metathesis, Ethenolysis, Vitamin A Derivatives

## Abstract

β-Carotene ethenolysis under promotion of well-defined ruthenium catalysts were examined as a novel method of synthesis of vitamin A derivatives. Efficient reaction was promoted by the second-generation Hoveyda catalyst. The products of ethenolysis in positions C15-C15′, C11-C12, and C9-C10 were detected, but cleavage of the C11-C12 double bond predominated. Even better regioselectivity at this position was observed for cross—metathesis between β-carotene and functionalized alkenes.

## INTRODUCTION

The important role of vitamin A in vision, bone growth, reproduction, and cell division and differentiation (Bates 1995) allows one to assume that its analogues may also be biologically active. For this reason there is a continuous need for new synthetic routes to such potentially active compounds. Retrosynthetic analysis reveals that β-carotene may serve as a cheap and easily available starting material for obtaining vitamin A analogues. The discovery of olefin metathesis and development of well-defined ruthenium and molybdenium alkylidene catalysts (Grubbs 2004; Connon and Blechert 2003) has provided a very powerful synthetic route to complex olefins. In this method new C–C double bond may be formed under very mild conditions and in a relatively short time, what is especially important for unstable substrates such as β-carotene.

In this paper the preliminary results of our studies on cross-metathesis (CM) of β-carotene with ethene (ethenolysis) (Kenneth et al. 2004; Forman et al. 2005) and other olefins are reported. To our best knowledge, the metathesis of β-carotene or other conjugated polyenes has not been explored so far, except 1,3-diene metathesis (Lee et al. 2003a, 2003b; Royer et al. 2003; Dewi et al. 2005; Ferrie et al. 2006, Moura-Letts and Curran 2007). However, ethenolysis of β-carotene required unprecedented cleavage of an internal double bond of the polyene system.

## METHOD

The products of β-carotene ethenolysis were rather difficult to predict. The characteristic for the β-carotene conjugated system, consisting of 11 double bonds, causes its CM to produce a complex mixture of compounds. However, the central C15-C15′ double bond was expected to be the most reactive one. Metathetic cleavage in this position would afford the desired retinol derivative. Other disubstituted double bonds (e.g., C7-C8 or C11-C12) seemed to be more hindered in the preferred all-*trans* conformation.

In the first experiments the β-carotene ethenolysis under promotion of various catalysts (Grubbs first generation, Grubbs first generation + phenol [Forman et al. 2005], Grubbs second generation, Hoveyda second generation) was examined. In a typical experiment, through a solution of β-carotene in dry toluene ethene was slowly bubbled, while a solution of a catalyst (20 mol%) in dry toluene was added dropwise within 15 min at rt. It was assumed that such amount of catalyst might be necessary because of possible chelation of ruthenium complex by polyene moiety. The second-generation Hoveyda catalyst appeared to be the only efficient promoter of β-carotene ethenolysis. Other catalysts proved completely ineffective.

As was expected ethenolysis afforded products with lower molecular weights than β-carotene. The GC/MS method with internal standard was employed to evaluate the reaction product distribution. For GC/MS analysis, aliquots of 1 μL ethenolysis mixture in dichloromethane (initial concentration of β-carotene in the reaction mixture was 1.86 mM) were prepared. Internal standard (nonadecane, 0.2 eq.) was added to each sample. Analytical conditions: GC/MS (EI) was carried out on a Perkin Elmer (AutoSystem XL) gas chromatograph coupled to the MS detector (Perkin Elmer TurboMass) in the electron impact mode (70 eV). The column was PE-5HT (30 m × 0.25 mm × 0.1 μm); carrier gas: helium (1 mL/min); the inlet mode was split: 50:1; and the injector temperature was 250°C. The initial column temperature was 60°C, and after 1 min the temperature was raised to 310°C with 4°C per min. Thereafter, the conditions were held for 6 min. The mass spectrometer measurement was scanned from 40 to 600 m/z. According to the literature, the GC/MS method was successfully applied in many cases to analyze products with lower molecular weight than retinol (Dunagin and Olesen 1964; Mordi et al. 1991, 1993; Sommerburg et al. 2003). The major metathesis products were hexaene **1** (product of C15-C15′ bond ethenolysis), tetraene **2** (product of C11-C12 bond ethenolysis), and triene **3** (product of C9-C10 bond ethenolysis) in the ratio of 1.3:1.5:0.1, as determined by GC/MS.

**SCHEME 1:**
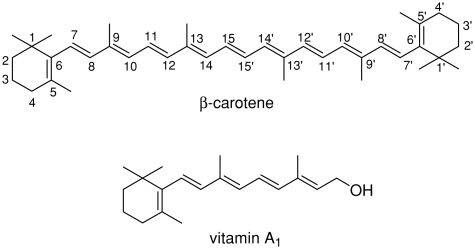


**SCHEME 2:**
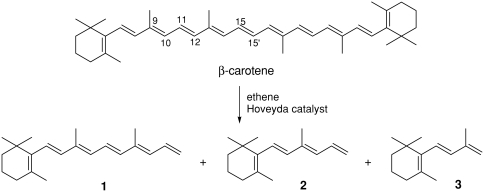


**SCHEME 3:**
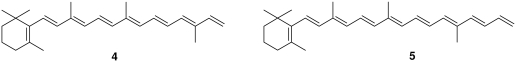


**SCHEME 4:**
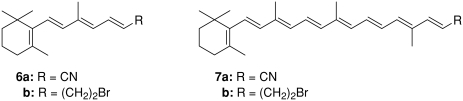


The partner products of cleavage in positions C11-C12 and C9-C10, namely octaene **4** and nonaene **5**, were not detected.

Presumably, these polyene products with higher molecular weight could not be eluted unchanged from the capillary column and therefore were not detectable using this technique. However, the method gave well-resolved chromatogram of lower-molecular-weight products.

## RESULTS

The control of ethenolysis reaction in time showed fast formation of tetraene **2**. This compound was detected immediately after adding the catalyst to the reaction mixture. The other two products **1** and **3** were detected only after 1 h. Contrary to expectations, the C11-C12 double bond seems to be the most preferred position for the carbene complex attack. This regioselectivity was also confirmed by cross-metathesis of β-carotene with other olefins: acrylonitrile or 4-bromo-but-1-ene in the presence of the second-generation Hoveyda catalyst. In both cases the major product, respectively, nitrile **6a** or bromide **6b**, was formed by cleavage in the position C11-C12. The partner product octaene **4** and possibly other CM products with higher molecular weight, for example, **7** were not detected, probably for reasons mentioned above.

## CONCLUSION

Further studies on qualitative and quantitative identification of products of β-carotene ethenolysis are in progress. The future investigations will include development of HPLC/UV or LC/MS methodologies preferable for determination of polyenes, as well as optimization of ethenolysis conditions. The CM reactions of β-carotene with functionalized alkenes seem to be even more promising than ethenolysis. Research in both these areas is ongoing in our laboratory.
